# Coaxially Electrospun 1D TiO_2_/Au_*x*_ (*X* = 2.5, 5.0, 7.5) Heterojunction Nanofibers: Unveiling the Role of TiO_2_–Au_*x*_ Interfaces in Carbon Monoxide Oxidation

**DOI:** 10.1021/acsmaterialsau.6c00151

**Published:** 2026-09-21

**Authors:** Balasubramanian Sriram, Sea-Fue Wang, Satoshi Kameoka, Ramakrishnan Vishnuraj

**Affiliations:** † Department of Materials and Mineral Resources Engineering, 34877 National Taipei University of Technology, No. 1, Section 3, Chung-Hsiao East Road, Taipei 106, Taiwan, ROC; ‡ Institute of Multidisciplinary Research for Advanced Materials, 92125 Tohoku University, 2-1-1 Katahira, Aoba-ku, Sendai 980-8577, Japan; § Center of Excellence in Advanced Materials and Green Technologies, Amrita School of Engineering Coimbatore, Amrita Vishwa Vidyapeetham, Coimbatore 641 112, India; ∥ Department of Chemical Engineering and Materials Science, Amrita School of Engineering Coimbatore, Amrita Vishwa Vidyapeetham, Coimbatore 641 112, India

**Keywords:** CO oxidation, TiO_2_/Au, nanofiber, coaxial electrospinning, greenhouse gas, sustainable

## Abstract

A “silent killer” that threatens aerobic life, carbon monoxide (CO) pollution has increased due to the Earth’s ecological equilibrium being upset by rapid industrialization and excessive resource extraction. In this study, titanium dioxide nanofibers (TiO_2_) decorated with gold nanoparticles (AuNPs@TiO_2_) were synthesized using a direct coaxial electrospinning technique, which eliminates the need for presynthesized AuNPs. The resulting AuNPs@TiO_2_ were evaluated for their catalytic performance in rapid CO oxidation. Catalytic activity was investigated for varying Au concentrations (2.5, 5.0, and 7.5 mM) incorporated into the TiO_2_ framework. The catalytic performance of TiO_2_–Au_*x*_ nanofibers varied with Au loading, with conversion efficiencies following the trend: TiO_2_/Au_0_ (673 K)<TiO_2_/Au_5.0_ (673 K)<TiO_2_/Au_7.5_ (623 K)<TiO_2_/Au_2.5_ (673 K). With its enhanced activity, Au_2.5_@TiO_2_ proves that the TiO_2_–Au interfacial contact is optimized at an ideal Au concentration, allowing for efficient charge transfer and oxygen activation. This study demonstrates a heterojunction-engineering strategy for developing a highly efficient catalytic CO oxidation, achieved through precise modulation of the TiO_2_–Au interfacial synergy to enhance electron transport and oxygen activation. This proposed approach aims to produce TiO_2_/Au for catalytic CO oxidation via coaxial electrospinning. It will provide insight into how designing the metal-oxide interface increases the kinetics of oxidation.

## Graphical abstract

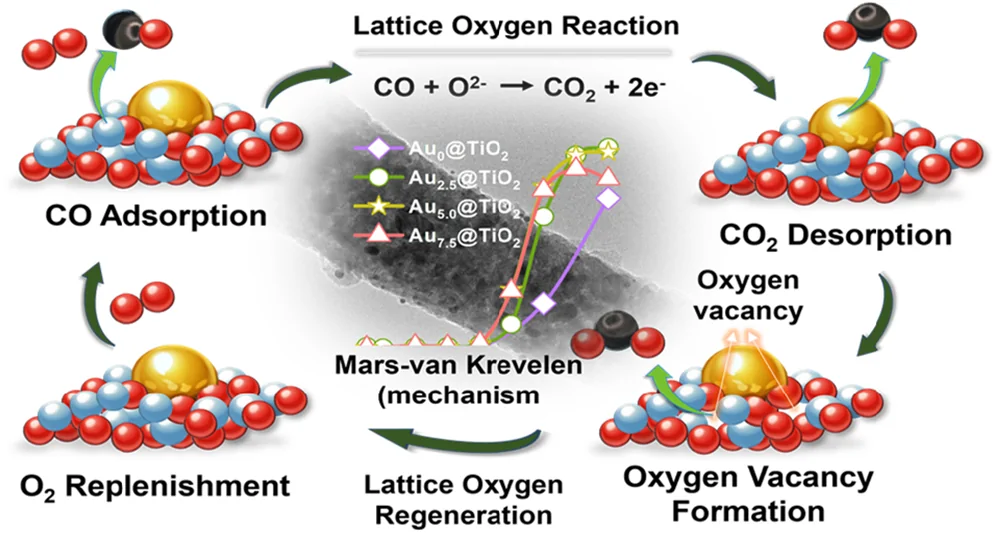

## Introduction

The escalating human reliance on fossil fuels has resulted in the emission of various toxic gases into the atmosphere, causing crucial environmental concerns.^1,2^ Amidst the myriads of hazardous air pollutants, “carbon monoxide (CO)” is the chief contributor to the bionetwork imbalance.^3,4^ According to reports, a concentration of 1.2% CO in the air can be lethal in just 3 min when exposed to normal atmospheric pressure.^5,6^ CO, as one of the common air pollutants, is harmful to humans and animals due to its stronger interaction with hemoglobin than oxygen gas (O_2_). During inhalation, it irreversibly binds to the Fe atom in hemoglobin (Hb), reducing oxygen transfer to body tissues.^7,8^ CO exposure reduces the oxygen-carrying capacity of Hb, leading to tissue hypoxia, poor cognitive function, brain damage, diminished vision, delayed reflexes, lethargy, and, in severe cases, death.^9–12^ CO is produced by incomplete combustion, and the most common sources of exposure include fire smoke inhalation, motor vehicles, and other engine exhaust.^11^ Mobile sources contribute significantly to CO emissions, necessitating rigorous environmental rules and the development of innovative catalytic technologies for emission control.^3,13^ Catalytic CO oxidation is the most successful strategy for CO removal, transforming toxic CO into comparatively innocuous CO_2_ (2CO + O_2_ = 2CO_2_) at low temperatures.^14^ The efficient catalytic conversion of CO, a highly toxic pollutant produced by incomplete combustion, into the less toxic and thermodynamically stable CO_2_ is fundamental to environmental purification, and this process is known as CO oxidation.^14^ In contrast to CO/CO_2_ hydrogenation, which is primarily focused on the utilization of carbon for value-added fuels and chemicals, CO oxidation is concerned with the efficient removal of CO from industrial emission streams and combustion emissions. Consequently, these strategies are combined rather than incompatible. In the process of catalysis, one of the most important stages is the heterogeneously catalyzed reaction of oxygen with CO.^15^ The development of catalytic materials that could simultaneously control CO emissions under oxygen-rich conditions is of great significance for the treatment of actual industrial flue gas.

A recent large-scale study was carried out to test the performance of platinum/alumina (Pt/Al_2_O_3_) catalysts in CO oxidation within the steel sector.^16^ These catalysts are well-known for their superior stability and activity in CO catalytic converters used in automobiles. Nevertheless, after roughly 2500 h of operation, the CO removal efficiency of Pt/Al_2_O_3_ decreased to below 40%, which indicates that it is not sufficiently stable over a prolonged period under conditions of manufacturing.^3^

As catalysts for low-temperature CO oxidation, transition metal oxides like manganese dioxide (MnO_2_),^17^ cobalt oxide (Co_3_O_4_),^18^ nickel oxide (NiO),^19^ copper oxide (CuO),^20^ and titanium dioxide (TiO_2_)^21,22^ have recently garnered a lot of interest. Strategies such as nanostructure engineering, heteroatom doping, and noble metal alteration have greatly improved their catalytic activity by creating effective heterostructured surfaces. In this context, Au nanoparticles (NPs) have predominantly demonstrated enhanced performance within the Au/TiO_2_ combination,^23^ where particle size is critical to achieving high catalytic activity.^24^ Recent developments involving in situ and operando spectroscopy have shown various molecular mechanisms controlling CO oxidation on Au/TiO_2_ catalysts.^25,26^ In order to facilitate the conversion of CO to CO_2_, it is assumed that the existence of dual active sites at the interface between Au and TiO_2_ plays a significant role.^27^ At low temperatures (−153 °C), CO molecules adsorbed on Ti^4+^ sites are responsible for low-temperature action, but bare TiO_2_ did not show any reactivity. It appears that the actual active centers are CO species attached to Ti^4+^ that are near Au nanoparticles. Additionally, CO adsorbed on Ti^4+^ demonstrated enhanced surface mobility relative to CO coupled with Au, and the calculated apparent activation energy for CO oxidation at Ti^4+^ sites was roughly 0.16 eV, which is considerably lower than the barrier for direct O_2_ activation on Au nanoparticles.^27,28^

TiO_2_ is usually produced by sol–gel,^29^ hydrolysis,^30^ hydrothermal,^31^ and electrospinning methods.^32,33^ Because of its unique advantages, electrospinning has become a popular method to develop heterogeneous catalysts. These include, among other things: (i) the ability to produce thin NFs/NPs with a high surface-to-volume ratio; (ii) the ability to functionalize NFs by simple blending; (iii) superior mechanical properties; and (iv) architectural versatility.^34^ A heterojunction of one-dimensional (1D) TiO_2_ NFs and 0-D AuNPs can significantly enhance the catalytic activity and interfacial electron transport.

Additionally, the coaxial design makes it possible to construct core–shell or heterojunction structures, which provide control over the placement of components and the contact between their surfaces. Even though it offers these benefits, not much research has been conducted into using it for developing catalytic materials, especially for gas-phase CO oxidation. Earlier studies on coaxially electrospun nanofibers have mostly concentrated on photocatalytic or energy-storage systems, whereas their use in heterogeneous gas-phase catalysis has been inadequately explored. In this study, we provide the coaxial electrospinning technique for the manufacture of one-dimensional TiO_2_/Au heterojunction nanofibers, ensuring close Au–TiO_2_ interfacial contact and uniform distribution of plasmonic Au nanoparticles. This one-of-a-kind structure makes it easier for electrons for movement and for active sites to be exposed, which makes the CO oxidation activity much better than that of catalysts made in the usual way.

In this work, the direct coaxial electrospinning of titanium dioxide nanofibers (TiO_2_ NFs) loaded with gold nanoparticles (AuNPs@TiO_2_ NFs) is presented for catalytic CO oxidation (Scheme 1). The effectiveness of CO oxidation catalysis was investigated by employing TiO_2_ nanostructures doped with varying amounts of Au_*x*_ (*X* = 2.5, 5.0, and 7.5 mM). The performance of TiO_2_–Au_*x*_ nanofibers in catalytic CO oxidation varied depending on the amount of Au loaded. The influence of the preparation method and the doped noble metal on the catalytic activity was assessed.

**Scheme 1:**
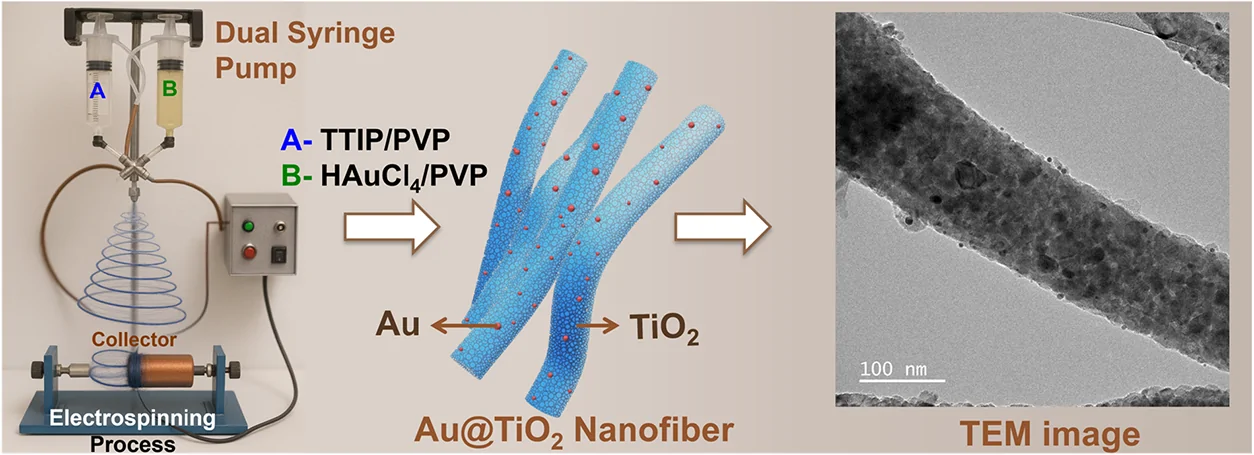
Schematic Illustration of Electrospinning Route Preparation of AuNPs@TiO_2_ NFs for Catalytic CO Oxidation

## Experimental Methods

### Preparation of AuNPs@TiO_2_ NFs

Coaxial electrospinning was employed to fabricate titanium dioxide nanofibers loaded with gold nanoparticles (AuNPs@TiO_2_ NFs). Initially, a poly­(vinylpyrrolidone) (PVP) solution (8% w/v) was prepared by dissolving 1.27 g of PVP in a solvent mixture containing 10 mL of dimethyl sulfoxide (DMF) and 5.8 mL of ethanol under magnetic stirring. The inner precursor solution (Solution A) was obtained by adding 6 mL of titanium isopropoxide (TTIP) to the PVP solution, followed by continuous stirring at room temperature for 2 h. The outer precursor solution (Solution B) was prepared by dissolving tetrachloroauric­(III) acid trihydrate (HAuCl_4_·3H_2_O) in 10% w/v PVP (10 mL DMF) and stirring vigorously at room temperature for 2 h to yield Au concentrations of 2.5, 5.0, and 7.5 mM. Both precursor solutions were loaded into separate syringes connected to a coaxial nozzle (core: 19 gauge; shell: 16 gauge). Electrospinning was performed at an applied voltage of 21 kV with a tip-to-collector distance of 18 cm. The flow rates of the core and shell solutions were maintained at 0.6 and 0.2 mL h^–1^, respectively, using individual syringe pumps. The as-spun fiber mats, comprising a TTIP/PVP core and an HAuCl_4_/PVP shell, were collected on a grounded metal plate. The mats were then calcined in air at 550 °C for 3 h at a heating rate of 5 °C min^–1^ to remove organic constituents and induce crystallization. During this process, HAuCl_4_ was thermally reduced to metallic Au, resulting in the formation of AuNPs uniformly distributed within the TiO_2_ nanofiber matrix. A schematic representation of the synthesis process is illustrated in Scheme 1.^32^

### Catalytic Measurements

The catalytic performance for CO oxidation was evaluated using a conventional fixed-bed flow reactor. The feed gas consisted of a mixture containing 0.998% CO and 0.500% O_2_, balanced with He. Approximately 100 mg of catalyst was loaded onto a bed of quartz wool inside a straight quartz tube with an inner diameter of about 6 mm. Catalytic tests were conducted over a temperature range of 298–772 K. The reaction products were analyzed using a Shimadzu GC-8A online gas chromatograph equipped with molecular sieve 5A columns for CO and O_2_ separation and Porapak Q columns for CO_2_ detection. Catalytic activity was determined from the percentage conversion of CO to CO_2_, calculated using eqs ([Disp-formula eq1]) and (2), after achieving a steady-state condition within 30 min of reaction time.

1 $$X_{\mathrm{CO}}\left(\%\right)=\frac{{CO}_{inlet}-{CO}_{outlet}}{{CO}_{inlet}}\times 100\%$$

2 $$X_{O_{2}}\left(\%\right)=\frac{O_{2inlet}-O_{2outlet}}{O_{2inlet}}\times 100\%$$

## Results and Discussion

### Morphological Analysis of Prepared AuNPs@TiO_2_

Field emission scanning electron microscopy (FESEM) and transmission electron microscopy (TEM) were employed to examine the morphology of the synthesized materials. As shown in Figure 1(a,b), the AuNPs@TiO_2_ nanofibers exhibit a one-dimensional fibrous architecture with rough surface texture and an average diameter of approximately 300 ± 50 nm. These observations were further supported by a low-resolution TEM (TEM) image of Au_2.5_@TiO_2_ (Figure 1(c)). The high-magnification (HRTEM) images of Au_0_@TiO_2_ (Figure 1(d–f)) clearly reveal that the absence of Au nanoparticles indicates pure TiO_2_. Lattice fringe analysis of the HRTEM image (Figure 1(f)) further revealed interplanar spacings of ∼0.327 nm and ∼0.357 nm for the rutile and anatase phases of TiO_2_, respectively. In a similar manner, the HRTEM image of Au_2.5_@TiO_2_ (Figure 1(g–i)) makes it abundantly evident that the nanoparticles of gold are dispersed evenly, as shown by the yellow circles. The selected area electron diffraction (SAED) pattern (Figure 1(h′)) displays distinct diffraction rings corresponding to the (101), (200), and (105) planes of TiO_2_ and the (111) plane of Au, confirming the successful formation of the AuNPs@TiO_2_ heterostructure. Additional results from the HRTEM lattice fringe analysis (Figure 1(i)) showed that the interplanar spacings for the (200) plane of face-centered cubic (*fcc*) Au were around ∼0.204 nm, whereas for the rutile and anatase phases of TiO_2_, the values were ∼0.327 nm and ∼0.357 nm, respectively. In Figure S1, the EDX spectrum of the Au@TiO_2_ NFs reveals the presence of all of the elements, including Ti, O, and Au. There is additional Cu present from the copper grid that was utilized for TEM.

**Figure 1:**
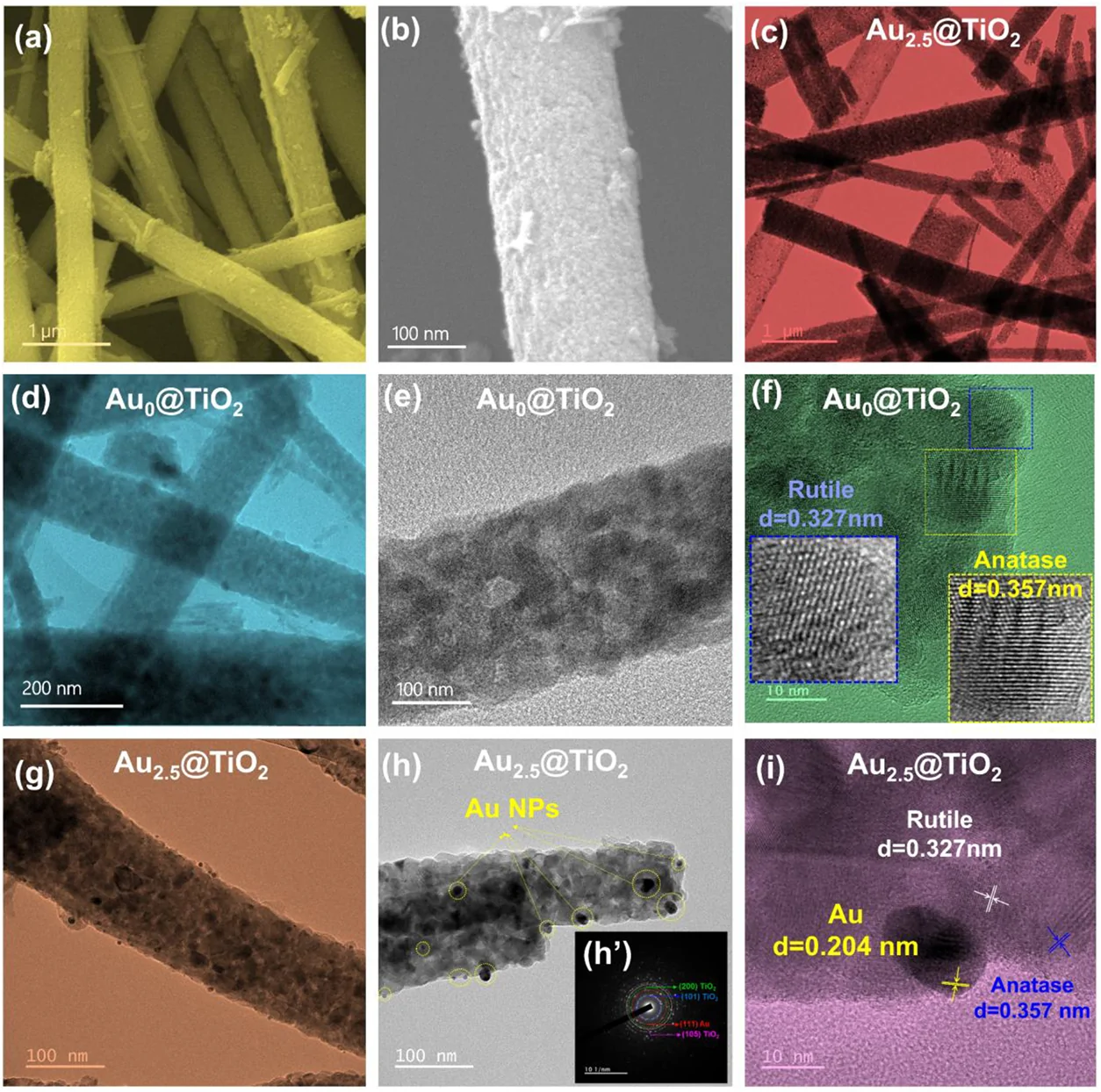
(a, b) FESEM and (c) TEM lower magnification images of in situ prepared Au_2.5_@TiO_2_ NFs. (d–f) HRTEM higher magnification images of Au_0_@TiO_2_ NFs with (f) lattice fringes of Au_0_@TiO_2_ NFs with rutile and anatase fringes. (g–i) HRTEM higher magnification images of Au_2.5_@TiO_2_ NFs with (h′) SAED patterns of Au_2.5_@TiO_2_ NFs. (i) Lattice fringes of Au_2.5_@TiO_2_ NFs with rutile and anatase fringes along with Au dopant.

### X-ray Diffraction (XRD) Analysis

X-ray diffraction (XRD) is a widely used nondestructive technique for determining the phase composition, crystallinity, and structural characteristics of nanomaterials. Figure 2(a) presents the XRD patterns of pure TiO_2_ nanofibers (NFs) and AuNPs@TiO_2_ NFs synthesized with varying Au precursor concentrations (2.5, 5.0, and 7.5 mM). The diffraction pattern of TiO_2_ NFs indicates a mixed-phase composition consisting of anatase (JCPDS Card No. 71–1166) and rutile (JCPDS Card No. 04–0551) phases, both exhibiting tetragonal crystal symmetry. The diffraction peaks corresponding to the anatase phase are indexed to the (101), (004), (200), (105), (211), (204), (116), (220), and (215) planes, appearing at 2θ values of 25.32°, 37.66°, 48.0°, 53.9°, 55.1°, 62.7°, 68.8°, 70.3°, and 75.0°, respectively. Meanwhile, the rutile phase is identified by diffraction peaks at 27.4°, 36.1°, 41.2°, 44.0°, 54.2°, 56.5°, 64.0°, 69.1°, 69.7°, and 76.5°, corresponding to the (110), (101), (111), (210), (211), (220), (310), (301), (112), and (202) planes, respectively. For the AuNPs@TiO_2_ NFs, in addition to the TiO_2_ peaks, new diffraction peaks appear at 38.0°, 44.2°, 64.5°, and 71.5°, corresponding to the (111), (200), (220), and (311) planes of face-centered cubic (fcc) Au (JCPDS Card No. 01–1174), confirming the successful incorporation of Au nanoparticles within the TiO_2_ matrix. Notably, a slight shift of the TiO_2_ diffraction peaks toward lower 2θ angles is observed with increasing Au content. This shift suggests a lattice expansion attributed to the interaction of Au with the TiO_2_ crystal lattice, implying partial strain or interfacial coupling between the metal and oxide phases.

**Figure 2:**
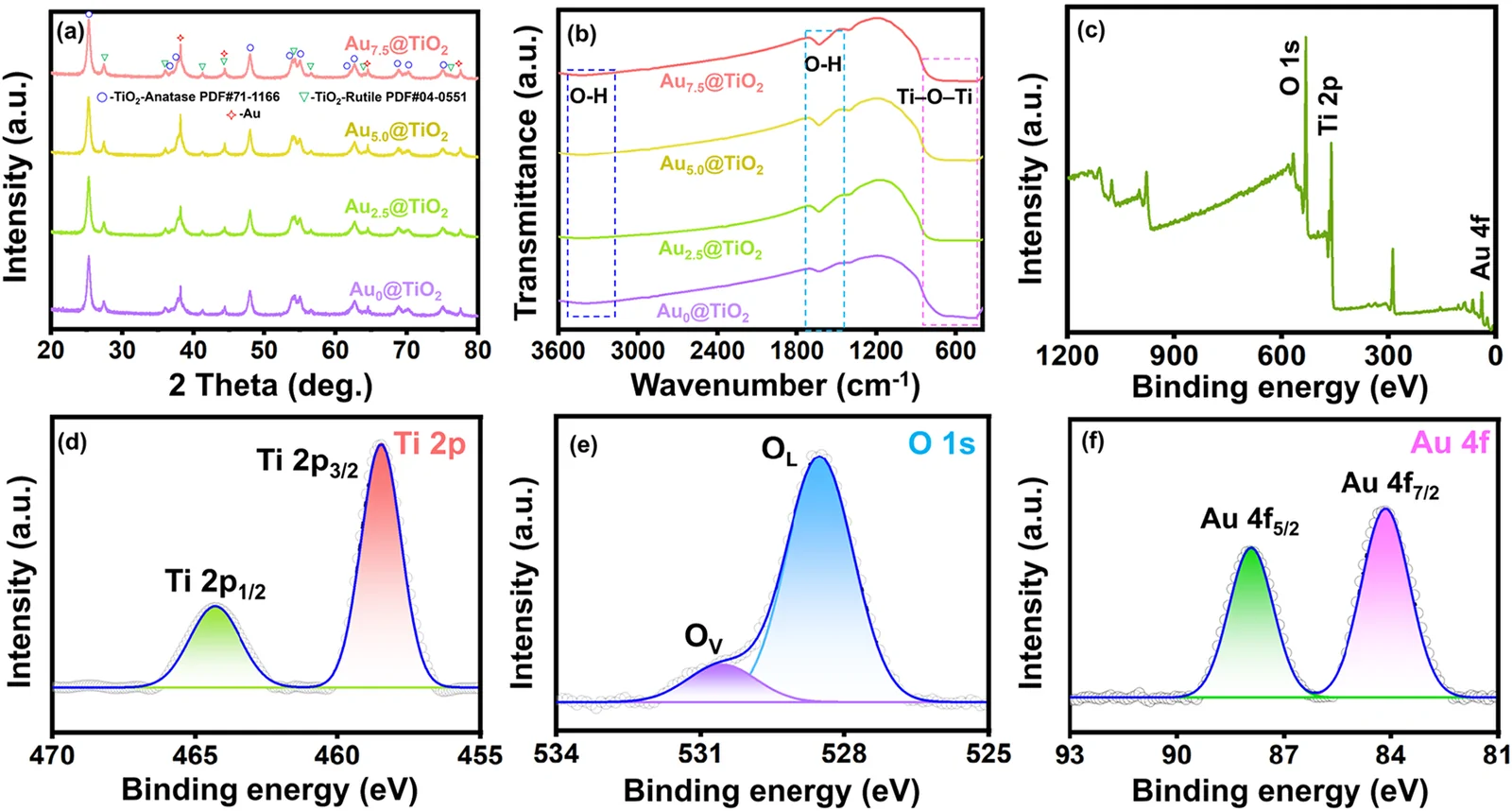
(a) XRD patterns and (a) FTIR spectra of TiO_2_ nanostructures doped with varying amounts of Au_x_ (Au_*x*_@TiO_2_, Au_2.5_@TiO_2_, Au_5.0_@TiO_2_, Au_7.0_@TiO_2_). XPS spectrum of Au_2.5_@TiO_2_: (c) survey spectrum, (d) Ti 2p, (e) O 1s, and (f) Au 4f.

### Fourier Transform Infrared Spectroscopy (FTIR) Analysis

Figure 2(b) illustrates the IR spectra of TiO_2_ nanostructures doped with varying amounts of gold (Au_*x*_@TiO_2_: Au_2.5_@TiO_2_, Au_5.0_@TiO_2_, Au_7.0_@TiO_2_). A dominant absorption band centered around 440–850 cm^–1^ is observed in all compositions, which is characteristic of Ti–O vibrations, confirming the formation of titanium dioxide. Furthermore, Au doping in TiO_2_ results in a slight broadening of the Ti–O–Ti bands within the 440–850 cm^–1^ region. This minor broadening arises from the modification of local bonding and electron density around Ti^4+^–O^2–^ sites, caused by lattice distortion due to Au incorporation. These findings are in good agreement with the XRD results.

### X-ray Photoelectron Spectroscopy (XPS)

The XPS survey spectrum of the Au_2.5_@TiO_2_ sample, shown in Figure 2(c), reveals the existence of Ti 2p, C 1s, O 1s, and Au 4f elements. In Figure 2(d), the high-resolution Ti 2p spectrum displays two distinct peaks that are attributed to Ti^4+^ 2p_3/2_ and Ti^4+^ 2p_1/2_. These peaks are centered at binding energies of ∼458.81 eV and ∼464.58 eV. The O 1s spectrum, which is observed in Figure 2(e), exhibits a dominant peak at ∼528.94 eV. This peak is attributable to lattice oxygen (O_L_) that corresponds with Ti–O bonds. In addition to merely adsorbed oxygen molecules, interior weakly coordinated oxygen ions, hydroxyl groups on the surface, and/or carbonate-related species, a secondary peak with a lower intensity was found at ∼530.28 eV. Figure 2(f) shows that the high-resolution spectrum of Au 4f exhibits two well-defined spin–orbit regions at binding energies of ∼83.97 eV and ∼88.01 eV, respectively. These components are indicative of the Au 4f_7/2_ and Au 4f_5/2_ levels. These characteristics unequivocally reveal that the lower intense of gold is found in the metallic (Au^0^) state.

### Raman Analysis

Figure S2 displays the Raman spectra of pure TiO_2_ NFs and Au_*x*_@TiO_2_ NFs with x values of 0, 2.5, 5.0, and 7.5 mM. The anatase phase of TiO_2_ is identified by the characteristic Raman bands at around 145 cm^–1^ (I-E_g_), 198 cm^–1^ (II-E_g_), 397 cm^–1^ (I-B_1g_), 512 cm^–1^ (A_1g_/II-B_1g_), and 638 cm^–1^ (III-E_g_). Modifications to the Raman peak strength and broadening of the characteristic bands after Au integration show that the TiO_2_ framework and Au nanoclusters interact strongly at the interface.

### BET Analysis Prereaction

The specific surface area of the synthesized catalysts plays a crucial role in determining their catalytic efficiency, as a higher active surface area provides more accessible active sites for CO oxidation. The Brunauer–Emmett–Teller (BET) surface area values of the prepared samples are summarized in Table S1. Among the samples, Au_7.5_@TiO_2_ exhibited the highest surface area (90.3 m^2^ g^–1^), followed by Au_5.0_@TiO_2_ (70.4 m^2^ g^–1^), Au_2.5_@TiO_2_ (56.8 m^2^ g^–1^), and Au_0_@TiO_2_ (55.0 m^2^ g^–1^). Although the surface area contributes significantly to catalytic activity, it alone does not fully account for the observed performance trends. Other factors, such as the degree of Au nanoparticle dispersion, particle morphology, and the interfacial contact between Au and TiO_2_, also play critical roles in influencing the overall CO oxidation kinetics.

### Catalytic Activity Assessment on CO Oxidation

The catalytic performance of the synthesized catalysts was evaluated for CO oxidation, as illustrated in Figures 3(a–d) and S3. Four samples with different Au loadings, Au_0_@TiO_2_, Au_2.5_@TiO_2_, Au_5.0_@TiO_2_, and Au_7.5_@TiO_2_, were examined. The incorporation of Au nanoparticles markedly enhanced the catalytic efficiency compared to pure TiO_2_, confirming the beneficial role of Au in promoting CO oxidation. Among all tested compositions, Au_2.5_@TiO_2_ exhibited the highest catalytic activity, achieving enhanced CO conversion at lower temperatures. TiO_2_ is mainly a reducible oxide that helps with O_2_ activation, oxygen migration, and vacancy replenishment by providing sites related to surface lattice oxygen and oxygen vacancies.^35^ On the other hand, Au nanoparticles provide the active sites for CO adsorption and activation by facilitating Au-CO interactions.^36^ Enhancing the electrical structure of the active sites and promoting O_2_ activation, the TiO_2_–Au interface generates interfacial charge redistribution through strong metal–support interactions.^37^ Rapid CO oxidation to CO_2_ occurs by the synergistic cooperation of CO adsorbed on Au and reactive oxygen species associated with TiO_2_. The primary contributions to CO adsorption/activation, lattice-oxygen participation/O_2_ activation, and interfacial charge transfer, respectively, are produced through Au, TiO_2_, and the TiO_2_–Au interface. A Schottky junction at the Au–TiO_2_ interface promotes electron redistribution from TiO_2_ to Au, resulting in an electron-rich Au surface and an electron-depleted TiO_2_ region. This allows a CO adsorption/activation on Au and O_2_ activation on TiO_2_. CO adsorbed on Au interacts with TiO_2_ to produce CO_2_ and oxygen vacancies, which are then substituted by gaseous O_2_, facilitating CO and O_2_ activation and increasing overall oxidation kinetics. These parameters collectively enhance the adsorption and activation of O_2_ molecules, thereby facilitating CO oxidation through an efficient interfacial electron transfer mechanism. The enhanced performance of Au_2.5_@TiO_2_ can be ascribed to the optimal Au–TiO_2_ interfacial synergy achieved via the coaxial electrospinning process, which promotes well-dispersed Au nanoparticles and strong metal–support interactions. This configuration effectively repositions surface atoms and maximizes charge transfer, establishing a robust platform for catalytic oxidation reactions.

**Figure 3:**
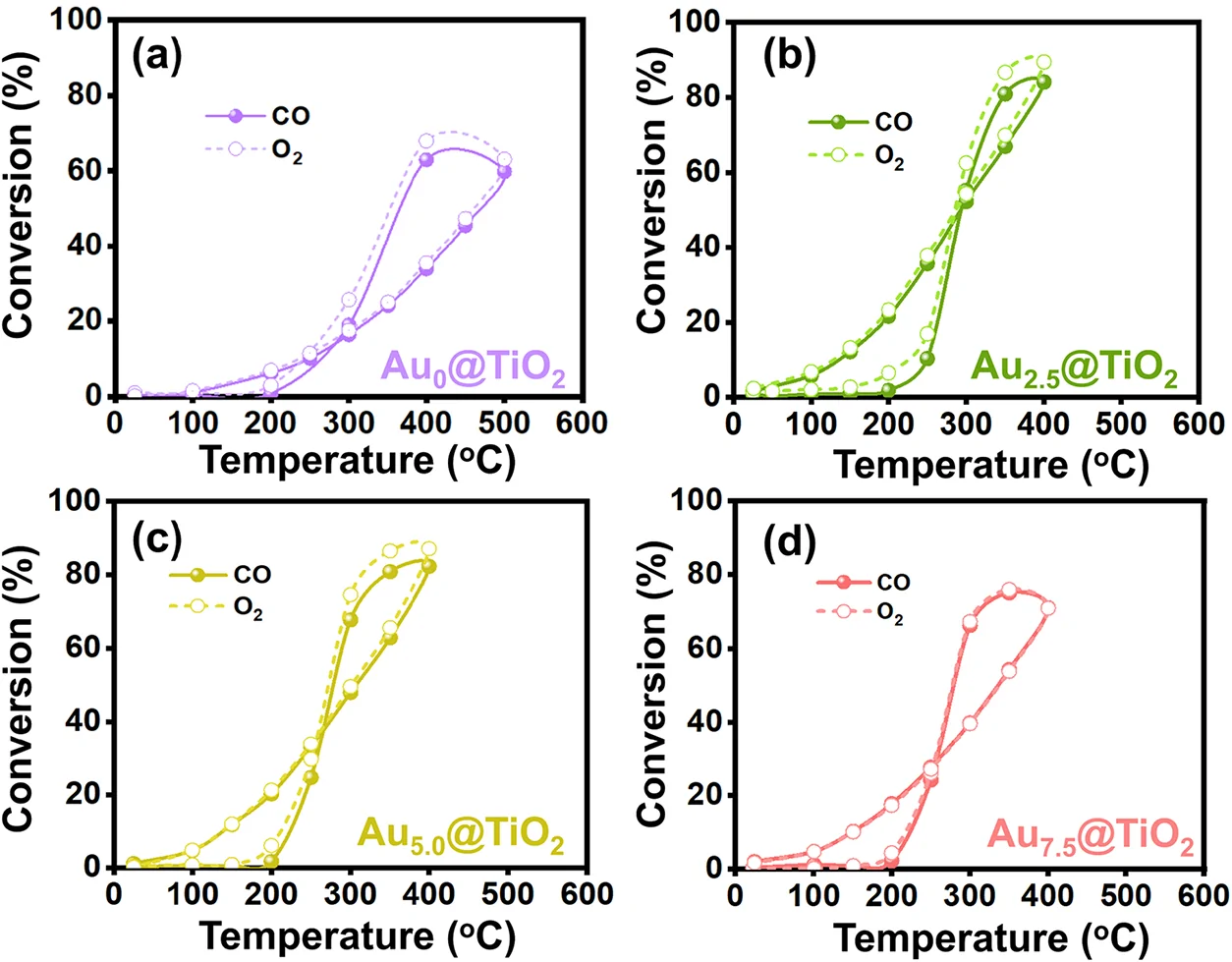
Catalytic activity of TiO_2_ nanostructures doped with varying amounts of Au_*x*_ ((a) Au_*x*_@TiO_2_, (b) Au_2.5_@TiO_2_, (c) Au_5.0_@TiO_2_, and (d) Au_7.5_@TiO_2_).

### Post-reaction XRD Analysis

X-ray diffraction (XRD) analysis was performed after the CO oxidation experiments to evaluate the structural stability and crystallinity of the catalysts. The postreaction XRD patterns of the samples are presented in Figure 4(a–d). All samples displayed diffraction profiles identical to those observed prior to the catalytic tests, with peaks consistent with their respective JCPDS reference cards. The diffraction pattern of TiO_2_ NFs reveals that they have a combined phase composition that includes both anatase (JCPDS Card No. 71–1166) and rutile (JCPDS Card No. 04–0551) phases. Both of these phases demonstrate tetragonal crystal structure symmetry according to the JCPDS numbers. In order, the anatase phase’s diffraction peaks can be found in planes (101), (004), (200), (105), (211), (204), (116), (220), and (215). They show up at 2θ values of ≈25.3°, 37.6°, 48.0°, 53.8°, 55.0°, 62.6°, 68.8°, 70.3°, and 75.0°, in order. Similarly, the rutile phase can be determined by its diffraction peaks at 27.3°, 36.1°, 41.1°, 44.0°, 54.1°, 56.5°, 64.0°, 69.1°, 69.7°, and 76.5°, which are the (110), (101), (111), (210), (211), (220), (310), (301), (112), and (202) planes, respectively. No significant changes, broadening, or removal of diffraction peaks were observed, showing that the catalysts’ lattice planes and crystal phases remained intact during the process. This discovery reveals that neither phase transition nor lattice deformation happened during CO oxidation, demonstrating the AuNPs@TiO_2_ NFs enhanced structural integrity and thermal stability under reaction circumstances. These findings demonstrate that the coaxially electrospun AuNPs@TiO_2_ catalysts maintain their crystallographic framework even after prolonged exposure to high-temperature oxidative environments, ensuring sustained catalytic durability.

**Figure 4:**
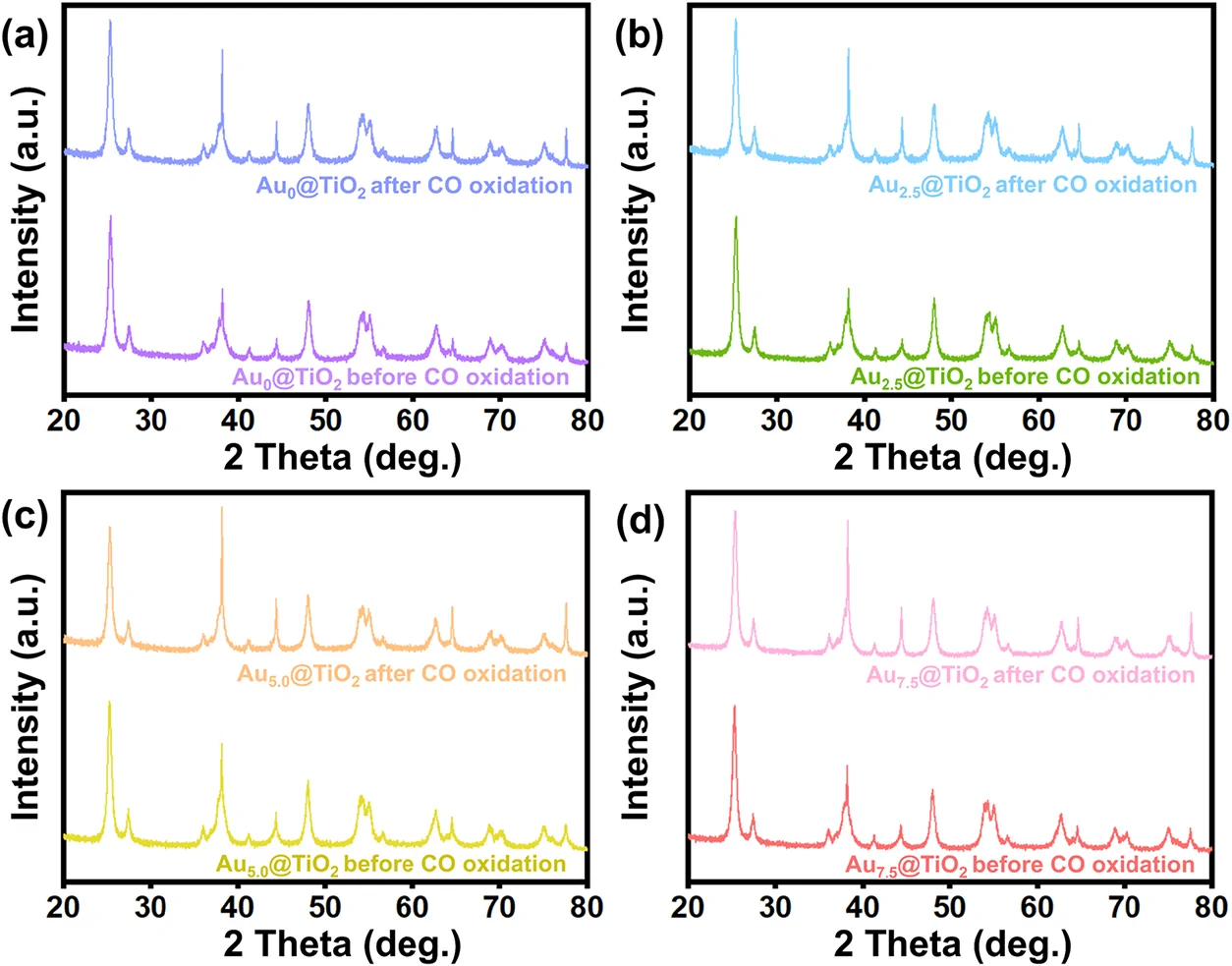
XRD patterns of (a) Au_*x*_@TiO_2_, (b) Au_2.5_@TiO_2_, (c) Au_5.0_@TiO_2_, and (d) Au_7.5_@TiO_2_ before and after the CO oxidation reactions.

### BET and TEM Analysis after CO Oxidation

To assess the catalyst stability following the CO oxidation reaction, the postreaction BET and TEM findings were compared to the catalytic performance. After the CO oxidation reaction, Brunauer–Emmett–Teller (BET BJH (Barrett–Joyner–Halenda) method) and HRTEM analysis were performed to evaluate changes in the surface characteristics of the catalysts, as shown in Figure 5(a–p), respectively. A noticeable increase in the specific surface area was observed across all compositions**:** Au_0_@TiO_2_, Au_2.5_@TiO_2_, Au_5.0_@TiO_2_, and Au_7.5_@TiO_2_, following the catalytic process.

**Figure 5:**
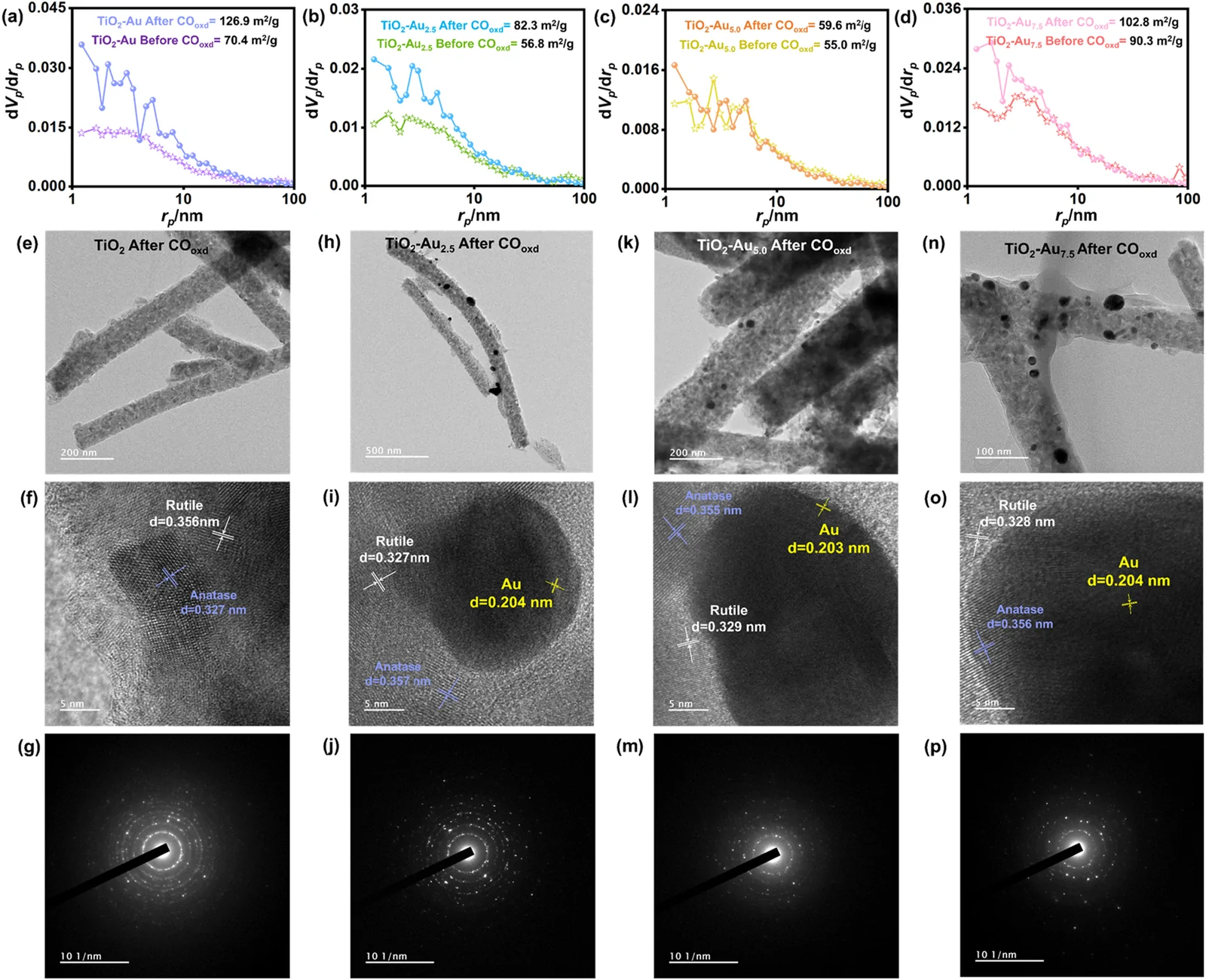
Mesopore distribution (BJH method) of (a) Au_*x*_@TiO_2_, (b) Au_2.5_@TiO_2_, (c) Au_5.0_@TiO_2_, and (d) Au_7.5_@TiO_2_ before and after the CO oxidation. HRTEM higher magnification images, lattice fringes, and SAED patterns of (e–g) Au_*x*_@TiO_2_, (h–j) Au_2.5_@TiO_2_, (k–m) Au_5.0_@TiO_2_, and (n–p) Au_7.5_@TiO_2_ after the CO oxidation, respectively.

The increase in BET surface area and apparent porosity of the Au_*x*_–TiO_2_ nanofibers after CO oxidation can be reasonably attributed to a combination of surface cleaning and mild nanostructural reorganization that proceeds without detectable changes in bulk crystallinity or fiber morphology, as evidenced by the essentially unchanged XRD patterns and retained 1D architectures in TEM/HRTEM images before and after reaction. Figure 5(e,h,k,n) shows that all of the nanofibers composed of TiO_2_, Au_2.5_@TiO_2_, Au_5.0_@TiO_2_, and Au_7.5_@TiO_2_ have a fibrous shape that is clearly defined, a surface texture that is rough, and an average diameter of around 300 ± 50 nm. These morphological traits are further supported by low-resolution TEM images. Figure 5f, a high-resolution TEM (HRTEM) image of pure TiO_2_, confirms the presence of AuNPs and the production of nanofibers devoid of impurities. The interplanar spacings of the rutile and anatase phases of TiO_2_ are about ≈0.356 and ≈0.327 nm, respectively, as shown by the detailed lattice fringe analysis. Similarly, the yellow markers in the HRTEM images of the Au_2.5_@TiO_2_, Au_5.0_@TiO_2_, and Au_7.5_@TiO_2_ NFs show that the AuNPs are evenly distributed across the surface of the TiO_2_ nanofiber. SAED patterns (Figure 5(g,j,m,p)) show distinct diffraction rings corresponding to TiO_2_ crystallographic planes (101, 200, and 105), as well as the typical (111) plane of metallic Au. The successful fabrication of the Au@TiO_2_ heterostructure nanofibers is confirmed by these diffraction characteristics, which also demonstrate the crystalline nature of both components. At elevated temperatures in a CO/O_2_ atmosphere, removing adsorbed reaction intermediates and residual surface species, along with continuous interfacial redox cycling at the Au–TiO_2_ interface, can expose previously inaccessible active sites and mesoporous domains. This results in a slight increase in the N_2_-accessible surface area while maintaining the overall structural framework. The good resistance to degradation of structure and nanoparticle aggregation during catalysis is further demonstrated by the postreaction TEM images, which further reveal that the one-dimensional nanofibrous shape and the homogeneous dispersion of Au nanoparticles are well preserved. Interestingly, the catalyst composed of Au_2.5_@TiO_2_ demonstrates the greatest CO oxidation activity, even though it does not have the higher BET surface area before or after the process. The catalytic efficacy is principally determined by the strong Au–TiO_2_ interfacial contact, efficient oxygen activation, and availability of reactive lattice oxygen, rather than total surface area alone.^38^ These findings show that the catalyst has good structural robustness and beneficial surface adaptability under reaction conditions, which is consistent with the Mars–van Krevelen (MvK) mechanism^39,40^ (Scheme 2). The Mars–van Krevelen (MvK) mechanism is directly employed in the oxidation of CO over Au–TiO_2_ catalysts, with TiO_2_ lattice oxygen playing a direct role in the reaction. One step in the catalyst surface oxygen vacancy generation process begins with CO adsorption on Au active sites, which is followed by a reaction with nearby TiO_2_ lattice oxygen to produce CO_2_. The final step of the catalytic cycle involves activating gaseous O_2_ molecules at the Au–TiO_2_ interface to restore the exhausted lattice oxygen.^41,42^ By boosting electron transfer and oxygen activation, the CO oxidation activity and stability are improved by the Au nanoparticles and the strong Au–TiO_2_ interaction, which also improves oxygen mobility and vacancy regeneration. The corresponding BET surface area values for all pre- and postreaction samples are summarized in Table S1.

**Scheme 2:**
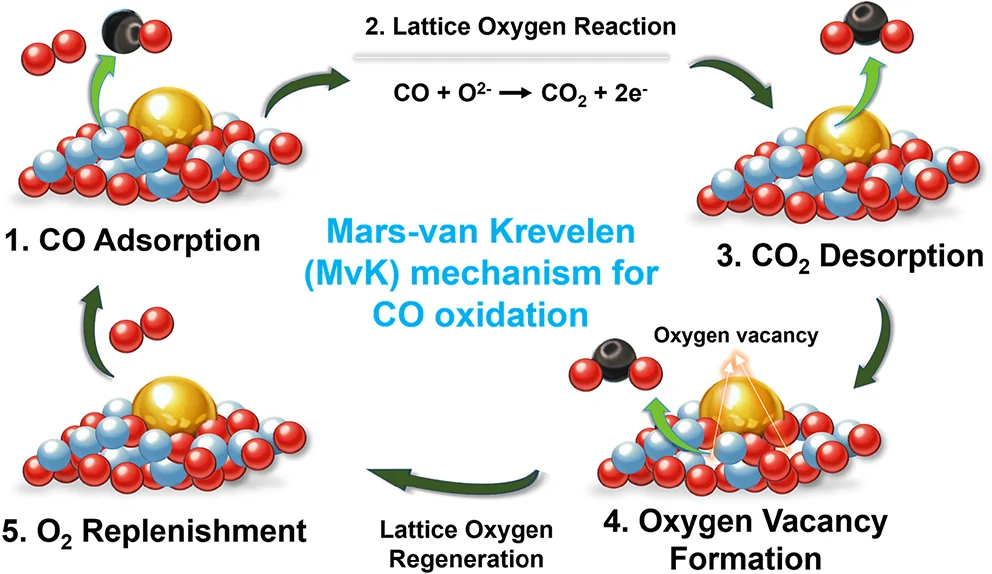
Schematic Representation of the Mars–Van Krevelen Process, Which Describes the Oxidation of Carbon Monoxide on the Au_*x*_–TiO_2_ Interfaces

## Conclusions

In this study, nanofiber catalysts with compositions Au_*x*_@TiO_2_ (*X* = 0, 2.5, 5.0, and 7.5 mM) were successfully synthesized using an in situ dual-syringe coaxial electrospinning technique. Among all of the prepared catalysts, Au_2.5_@TiO_2_ exhibited the highest catalytic efficiency, achieving the maximum CO conversion at 673.15 K. The enhanced CO oxidation performance of Au_2.5_@TiO_2_ can be explained by a Mars–van Krevelen-type mechanism, wherein CO molecules react with lattice oxygen at the Au–TiO_2_ interface to form CO_2_. The resulting oxygen vacancies are subsequently replenished by gaseous O_2_, maintaining continuous catalytic turnover. The strong interfacial coupling between Au and TiO_2_ facilitates efficient electron transfer and enhances the adsorption and activation of both CO and O_2_ molecules, thereby accelerating the overall reaction kinetics. This investigation provides valuable insight into the structure–activity relationship governing Au–TiO_2_ heterostructures and establishes a robust framework for designing efficient AuNPs@TiO_2_ nanofiber catalysts for low-temperature pollutant oxidation in exhaust treatment and environmental remediation applications.

## Supplementary Material



## Notes

Detailed materials, reagents, instrumentation, and methods. EDX spectrum of Au_*x*_@TiO_2_ NFs. Raman spectra of all TiO_2_@Au_*x*_ samples. CO conversion of the heating process of all Au_*x*_@TiO_2_ samples. BET specific surface area of all of the samples before CO oxidation and after CO oxidation (PDF)
