# Supplementary material for: Coaxially Electrospun 1D TiO2/Au x (X = 2.5, 5.0, 7.5) Heterojunction Nanofibers: Unveiling the Role of TiO2–Au x Interfaces in Carbon Monoxide Oxidation
Source: ACS Mater Au. 2026 Sep 21;6(6):1504–12. doi: 10.1021/acsmaterialsau.6c00151 (PMC13760635; doi:10.1021/acsmaterialsau.6c00151)
Supplement: Supplementary file 1 [file mg6c00151_si_001.pdf]

Supporting information for

Coaxially Electrospun 1D TiO_2/Au_x (X=2.5, 5.0, 7.5) Heterojunction Nanofibers: Unveiling the Role of TiO_2–Au_x Interfaces in Carbon Monoxide Oxidation

Balasubramanian Sriram[a], Sea-Fue Wang[a,], Satoshi Kameoka[b], Ramakrishnan Vishnuraj[c,d]*

[a]*Department of Materials and Mineral Resources Engineering, National Taipei University of Technology, No. 1, Section 3, Chung-Hsiao East Road, Taipei 106, Taiwan, ROC.*

[b]*Institute of Multidisciplinary Research for Advanced Materials, Tohoku University, 2-1-1 Katahira, Aoba-ku, Sendai, 980-8577, Japan.*

[c]*Center of Excellence in Advanced Materials and Green Technologies, Amrita School of Engineering Coimbatore, Amrita Vishwa Vidyapeetham, Coimbatore- 641 112, India.*

[d]*Department of Chemical Engineering and Materials Science, Amrita School of Engineering Coimbatore, Amrita Vishwa Vidyapeetham, Coimbatore- 641 112, India.*

Corresponding author:

Dr. S.F. Wang: sfwang@ntut.edu.tw

Chemicals and materials

Chloroauric acid trihydrate ($HAuCl_4 \cdot 3H_2O$), titanium isopropoxide ($Ti[OCH(CH_3)_2]_4$ (TTIP)), polyvinylpyrrolidone (PVP, Mw = 2,00,000), acetic acid (AA), dimethylformamide (DMF), ethanol, were purchased from Sigma-Aldrich (Taiwan). All chemicals used in the experiments are of analytical purity.

Instrumentation and methods

Phase configuration is identified using X-ray diffraction analysis (XRD) (D2 PHASER, Bruker (XRD) instrument. The microstructure of samples are studied utilizing field emission scanning electron microscopy (FESEM; JEOL-6500F) and high resolution (HR) transmission electron microscopy (TEM) (JEOL JEM-2100F (HR)) operating at 200 kV attached with selected area electron diffraction (SAED) pattern. The functional groups of the synthesized materials were analyzed by Fourier Transform Infrared Spectroscopy (FT-IR) technique, (FT-IR spectrophotometer, Shimadzu, IR Tracer 100). Brunauer-Emmett-Teller (BET) analysis utilized (BEL SORP-mini). By utilizing these characterization methods, the physical properties of the as-prepared materials are investigated. The reaction products were monitored by an on-line gas chromatograph (Shimadzu GC-8A) equipped with molecular sieve 5A (O_2, CO) and Porapak Q (CO_2) columns.

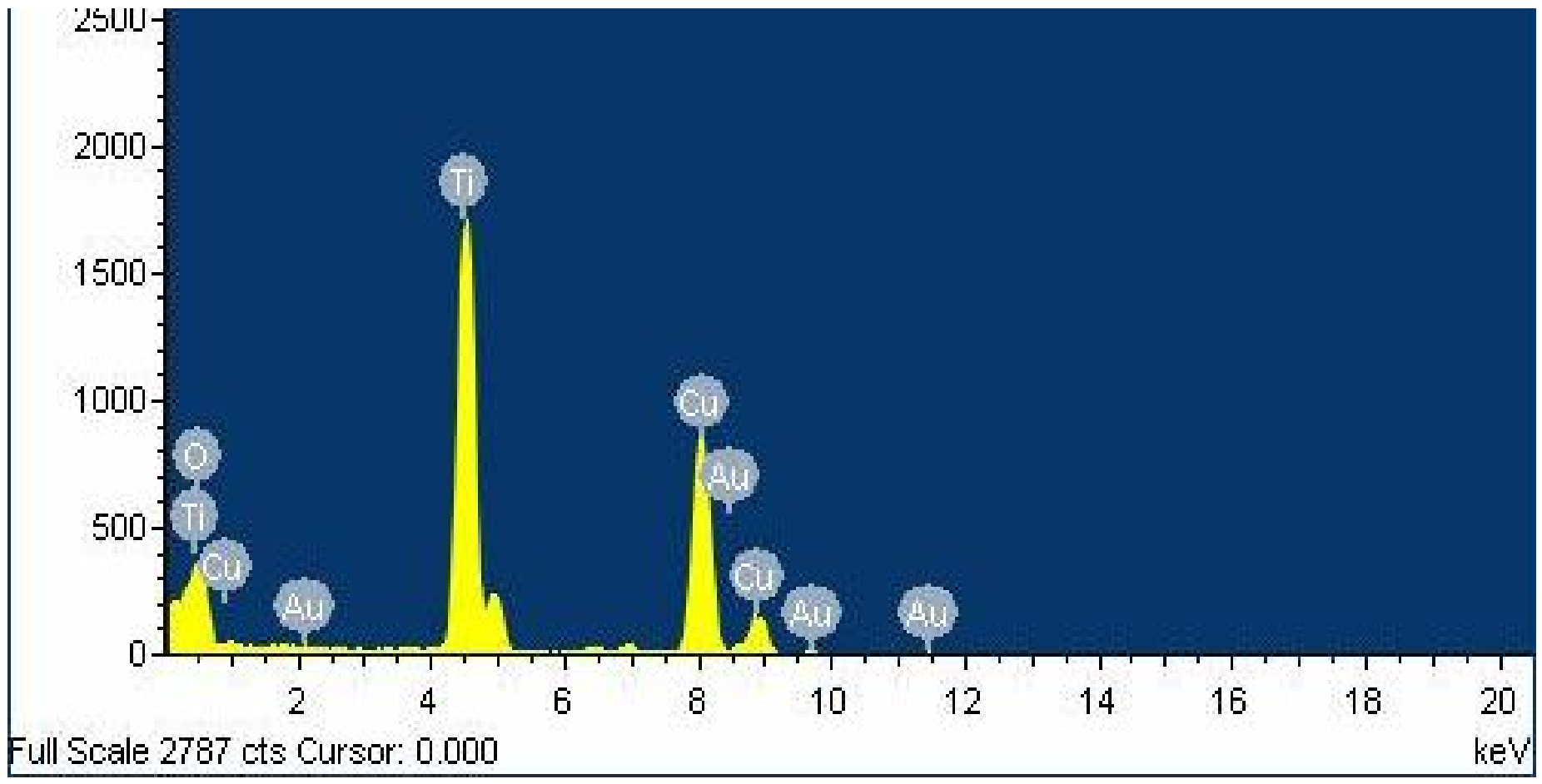

Figure S1. EDX spectrum of $Au_x@TiO_2$ NFs.

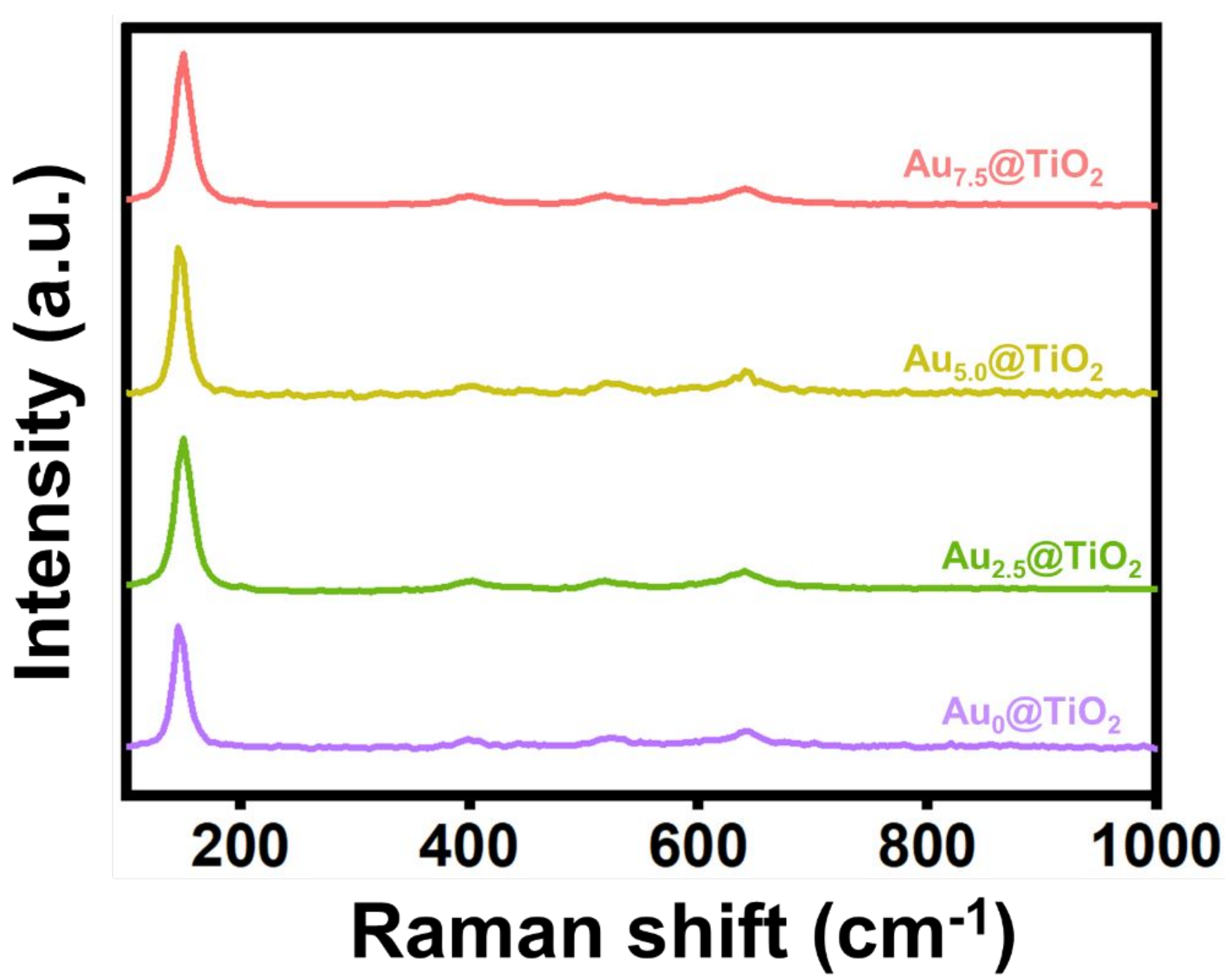

Figure S2. Raman spectra's of TiO_2 nanostructures doped with varying amounts of Au_x ($Au_x@TiO_2$, $Au_{2.5}@TiO_2$, $Au_{5.0}@TiO_2$, $Au_{7.0}@TiO_2$)

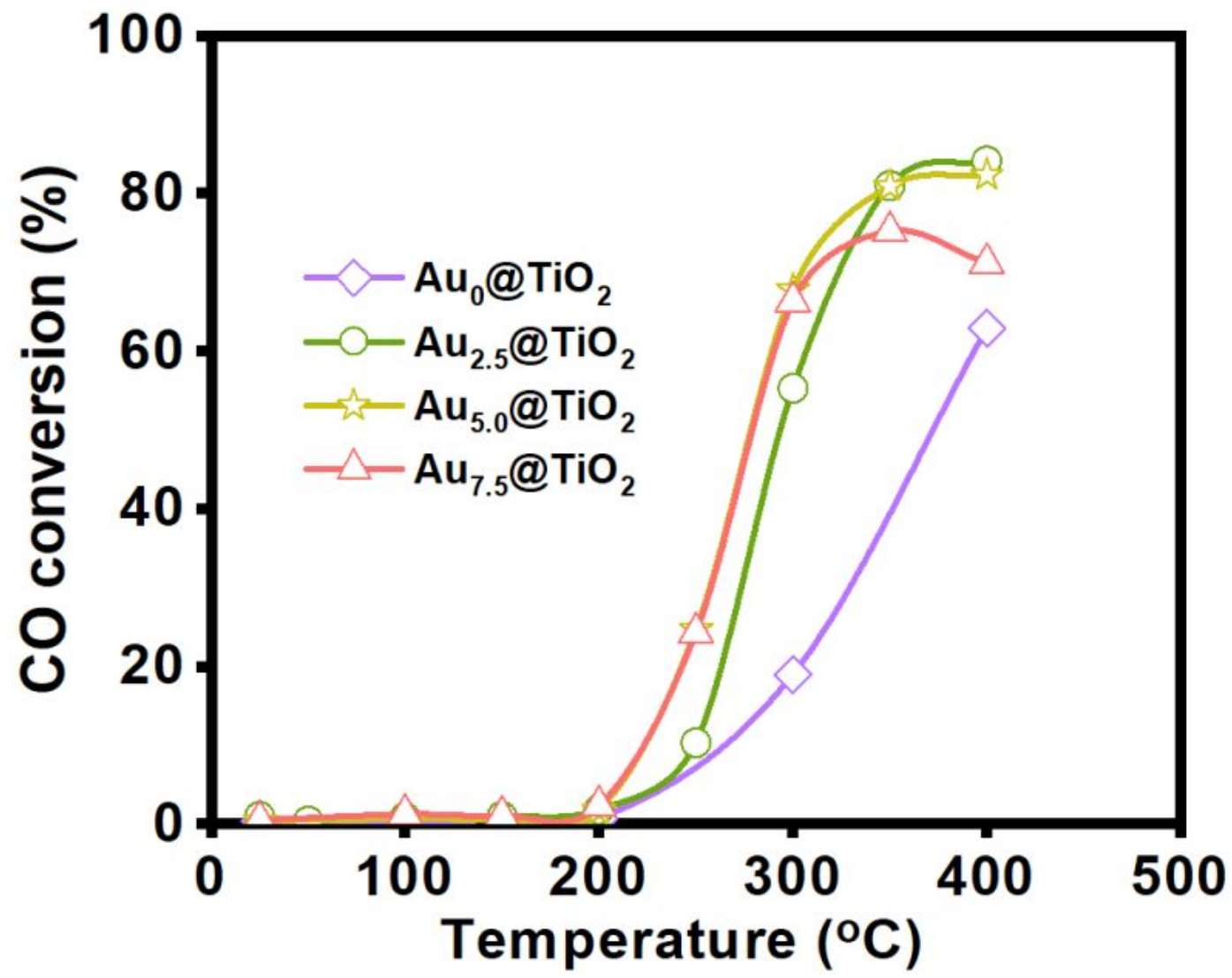

Figure S3. Catalytic CO conversion of heating process of $Au_x@TiO_2$: $Au_0@TiO_2$, $Au_{2.5}@TiO_2$, $Au_{5.0}@TiO_2$, $Au_{7.0}@TiO_2$.

Table S1. BET specific surface area of $Au_x@TiO_2$: $Au_{2.5}@TiO_2$, $Au_{5.0}@TiO_2$, $Au_{7.0}@TiO_2$ before CO oxidation and after CO oxidation. (Adsorbate: N_2 Pretreat in evac. 150°C/1 h).

**Samples after HyTL**	**S_{BET} (m^2/g)**	
	**Before CO oxidation**	**After CO oxidation**
$Au_0@TiO_2$	70.4	126.9
$Au_{2.5}@TiO_2$	56.8	82.3
$Au_{5.0}@TiO_2$	55.0	59.6
$Au_{7.5}@TiO_2$	90.3	102.3
